# Aldosterone Synthase Inhibitors: Emerging Therapeutic Strategies in Resistant Hypertension

**DOI:** 10.3390/life16091495

**Published:** 2026-09-07

**Authors:** Akshyaya Pradhan, Monika Bhandari, Abhishek Singh, Pravesh Vishwakarma, Kunal Mahajan, Marco Alfonso Perrone, Akash Batta

**Affiliations:** 1 Department of Cardiology, King George’s Medical University, Shahmina Road, Chowk, Lucknow 226003, Uttar Pradesh, India; akshyaya33@gmail.com (A.P.); drmonikab@gmail.com (M.B.); docabhishek23@gmail.com (A.S.); dr.pvishwakarma@gmail.com (P.V.); 2 Department of Cardiology, Himachal Heart Institute, Mandi 175021, Himachal Pradesh, India; kunalmahajan442@gmail.com; 3 Division of Cardiology and CardioLab, Department of Clinical Sciences and Translational Medicine, University of Rome Tor Vergata, 00133 Rome, Italy; marco.perrone@uniroma2.it; 4 Department of Cardiology, Dayanand Medical College and Hospital, Ludhiana 141001, Punjab, India

**Keywords:** aldosterone, antihypertensive agents, chronic kidney disease, CYP11B2, heart failure, hyperaldosteronism, hypertension, mineralocorticoid receptor antagonists, primary aldosteronism, renin-angiotensin system, resistant hypertension

## Abstract

Resistant hypertension (RH) is a high-risk phenotype associated with increased cardiovascular and renal morbidity despite multidrug therapy. Dysregulation of the renin–angiotensin–aldosterone system (RAAS), particularly excess aldosterone activity, plays a central role in the pathophysiology of RH. Although conventional RAAS-targeted therapies including angiotensin-converting enzyme inhibitors, angiotensin receptor blockers, and mineralocorticoid receptor antagonists improve outcomes, their effectiveness is limited by aldosterone breakthrough, persistent non-genomic aldosterone effects, hyperkalaemia, and off-target adverse effects. Aldosterone synthase inhibitors (ASIs) have emerged as a novel therapeutic strategy targeting CYP11B2, the terminal enzyme responsible for aldosterone biosynthesis. This review summarises the physiological basis of aldosterone synthesis, the pathological consequences of aldosterone excess, and the pharmacological evolution of ASIs. Early-generation agents were limited by inadequate selectivity between CYP11B2 and the closely related CYP11B1 enzyme, resulting in cortisol suppression and deoxycorticosterone accumulation. Advances in structural biology and medicinal chemistry enabled the development of second-generation ASIs with markedly improved selectivity and preserved cortisol biosynthesis. Recent clinical trials of baxdrostat, lorundrostat, vicadrostat, and dexfadrostat have demonstrated clinically meaningful reductions in blood pressure and albuminuria with acceptable safety profiles. Based on the positive trial data, baxdrostat has become the first in class ASI to be approved by regulatory authorities for management of uncontrolled hypertension.

## 1. Introduction

Resistant hypertension (RH) is a clinically significant and high-risk phenotype of hypertension, defined as blood pressure that remains above target despite adherence to treatment with at least three antihypertensive agents of different classes, including a diuretic, at maximally tolerated doses, or controlled blood pressure requiring four or more medications [1]. Hypertension affects over 1.3 billion individuals worldwide and remains the leading modifiable contributor to global cardiovascular morbidity and mortality [2]. Within this population, resistant hypertension is estimated to occur in approximately 10–20% of treated hypertensive patients [1]. However, after exclusion factors such as poor medication adherence, white-coat effect, and inaccurate blood pressure measurement, the true prevalence of RH is closer to 5–10% [3]. A large meta-analysis involving more than 3.2 million individuals reported a pooled prevalence of apparent RH of 14.7% and true RH of approximately 10.3%, underscoring its substantial global burden [3]. The prevalence is significantly higher among high-risk populations, including patients with chronic kidney disease (CKD), diabetes mellitus, obesity, and older age, in whom rates may exceed 20% [3,4]. Importantly, RH is associated with a markedly increased risk of adverse cardiovascular outcomes, including stroke, coronary artery disease, heart failure, and all-cause mortality, reflecting its characterisation as a distinct and particularly severe hypertensive phenotype [5].

Against this background, dysregulation of the renin–angiotensin–aldosterone system (RAAS) represents a central mechanistic pathway underpinning cardiorenal disease and remains a major therapeutic target across both conventional and emerging treatment strategies [4,6]. Aldosterone, a key effector hormone of the RAAS synthesised in the adrenal cortex, exerts critical effects on sodium and water homeostasis, blood pressure regulation, and adverse cardiovascular remodelling [7]. Pharmacologic inhibition of the RAAS using angiotensin-converting enzyme inhibitors, angiotensin receptor blockers, and mineralocorticoid receptor antagonists is associated with substantial improvements in clinical outcomes across hypertension, CKD, and heart failure [8,9]. Unlike earlier reviews focused primarily on pharmacological development or pooled blood-pressure efficacy, this review integrates the evolution of CYP11B2 selectivity with contemporary phase II/III evidence, regulatory developments, practical clinical considerations, and the emerging role of ASIs across resistant hypertension, primary aldosteronism, CKD, and heart failure.

## 2. Review Methodology

Our article is a narrative review which explore the potential of aldosterone synthase inhibitors (ASIs) as an emerging therapeutic option in RH. A targeted literature search was used to identify mechanistic studies, randomised clinical trials, systematic reviews and meta-analyses, clinical-trial registry records, contemporary guidelines, and regulatory documents relevant to aldosterone synthase inhibition. PubMed/MEDLINE, Embase, Scopus, Web of Science, and ClinicalTrials.gov were searched using combinations of “aldosterone synthase inhibitor”, “CYP11B2”, “resistant hypertension”, “baxdrostat”, “lorundrostat”, “vicadrostat”, “dexfadrostat”, “osilodrostat”, “primary aldosteronism”, “chronic kidney disease”, and “heart failure”. The literature was updated through 15 August 2026. Peer-reviewed phase II/III trials were prioritised for efficacy and safety claims; conference abstracts, press releases, and other non-peer-reviewed sources were retained only when no full publication was available and were clearly identified as preliminary. No formal risk-of-bias assessment or quantitative meta-analysis was performed because the purpose was a narrative synthesis rather than a systematic review.

## 3. The Renin–Angiotensin–Aldosterone System: A Brief Overview

The RAAS is a highly conserved neurohumoral cascade that plays a central role in the regulation of systemic blood pressure, intravascular volume, and electrolyte balance [10,11]. Under physiological conditions, RAAS activity is tightly controlled through a dynamic feedback mechanism responsive to changes in renal perfusion pressure, sodium delivery to the macula densa, and sympathetic nervous system activation. However, persistent dysregulation and overactivation of this system are now widely recognised as key pathophysiological contributors to several major cardiovascular and renal disorders, including hypertension, heart failure, chronic kidney disease (CKD), and primary aldosteronism, collectively affecting over one billion individuals worldwide [12,13].

The RAAS cascade is initiated by the liver, which constitutively synthesises and releases angiotensinogen, an alpha-2 glycoprotein, into the circulation [14]. In response to reduced renal perfusion, decreased sodium chloride delivery to the macula densa, or β1-adrenergic stimulation, specialised granular cells within the juxtaglomerular apparatus of the kidney secrete renin, an aspartyl protease [15]. Renin cleaves angiotensinogen to form angiotensin I (Ang I), a biologically inactive decapeptide [16]. Ang I is subsequently converted into angiotensin II [Ang II], the principal effector peptide of the system, by angiotensin-converting enzyme (ACE), which is predominantly expressed on the luminal surface of pulmonary vascular endothelium, as well as on systemic and renal endothelial cells [17]. In addition to ACE-dependent conversion, alternative enzymatic pathways, such as those involving chymase, cathepsin G, and tonin also contribute to Ang II generation, particularly within cardiac and vascular tissues. These non-ACE pathways may partly explain the incomplete suppression of RAAS activity observed with ACE inhibitor therapy [18].

Angiotensin II mediates its primary biological effects predominantly through the angiotensin II type 1 (AT1) receptor, a G-protein-coupled receptor expressed across multiple organ systems, including vascular smooth muscle, adrenal cortex, kidney, heart, and the central nervous system [19]. Activation of the AT1 receptor promotes vasoconstriction, enhances renal sodium and water reabsorption, augments sympathetic activity, and stimulates aldosterone synthesis and secretion from the zona glomerulosa of the adrenal cortex [20]. In contrast, a counter-regulatory arm of the RAAS exists, mediated by the angiotensin II type 2 (AT2) receptor and the ACE2–angiotensin-[1–7]–Mas receptor axis, which generally exert vasodilatory, natriuretic, and anti-fibrotic effects [21].

A comprehensive understanding of the RAAS framework provides important insight into therapeutic strategies targeting this system. Selective inhibition of aldosterone synthesis at its terminal enzymatic step may offer a more focused approach compared with upstream blockade, which affects the cascade more broadly.

### 3.1. Aldosterone Biosynthesis: The Steroidogenic Pathway in the Zona Glomerulosa

Aldosterone biosynthesis occurs exclusively within the zona glomerulosa, the outermost layer of the adrenal cortex, through a sequence of tightly regulated mitochondrial and microsomal enzymatic reactions that convert cholesterol, the universal precursor of all steroid hormones, into the principal mineralocorticoid [22,23]. A key feature of this process is the zone-specific expression of the terminal enzyme, CYP11B2 (aldosterone synthase), which is restricted to the zona glomerulosa. This contrasts with the adjacent zona fasciculata, where the closely related enzyme CYP11B1 is expressed. Such anatomical and enzymatic specificity underlies the relative selectivity of aldosterone synthesis and provides a rationale for targeted pharmacological intervention [24].

The initial and rate-limiting step of adrenal steroidogenesis involves the transport of cholesterol from cytoplasmic lipid stores to the inner mitochondrial membrane. This process is mediated by the steroidogenic acute regulatory protein (StAR) and is acutely regulated by angiotensin II, extracellular potassium, and adrenocorticotropic hormone (ACTH) [25]. Within the mitochondria, cholesterol undergoes side-chain cleavage by CYP11A1 (P450scc) to generate pregnenolone, the common precursor for all adrenal steroid hormones [26]. Pregnenolone is subsequently transferred to the endoplasmic reticulum, where it is converted to progesterone by 3β-hydroxysteroid dehydrogenase type 2 (3β-HSD2), followed by 21-hydroxylation via CYP21A2 to form 11-deoxycorticosterone (DOC) [27].

Although DOC possesses relatively weak mineralocorticoid activity, it can activate the mineralocorticoid receptor at elevated concentrations, contributing to sodium retention. This property has particular relevance in pharmacological contexts, including the development of early ASIs, as discussed further [28].

DOC then re-enters the mitochondria, where it is converted to corticosterone through 11β-hydroxylation mediated by CYP11B1. In humans, corticosterone serves primarily as a precursor in mineralocorticoid synthesis [29]. Within the zona glomerulosa, it undergoes further transformation by CYP11B2, which catalyses a series of sequential oxidation reactions culminating in the formation of aldosterone [30,31]. These reactions occur at a single enzymatic site, making CYP11B2 the exclusive and non-redundant determinant of aldosterone production. To date, no alternative pathway for de novo aldosterone synthesis has been identified in human adrenal tissue, reinforcing its role as a discrete therapeutic target [32].

**Figure 1:**
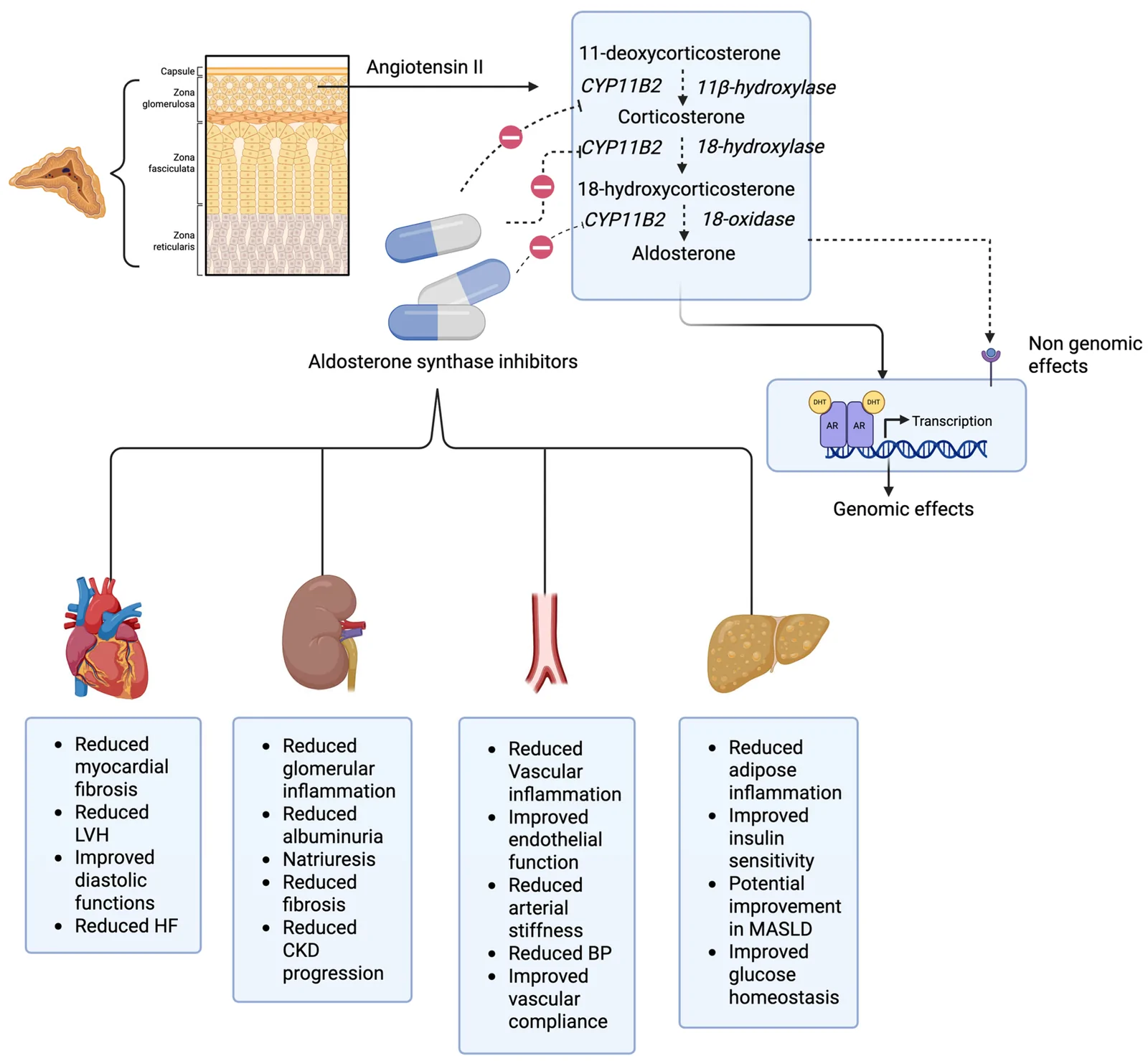
Multisystem mechanisms and therapeutic impact of aldosterone synthase inhibitors across the cardiovascular–renal–metabolic axis. Aldosterone synthase inhibitors selectively inhibit aldosterone synthase (CYP11B2) within the zona glomerulosa of the adrenal cortex, thereby reducing aldosterone biosynthesis while exerting minimal effects on cortisol production. Decreased aldosterone availability attenuates mineralocorticoid receptor activation, resulting in favourable cardiovascular, renal, and metabolic effects. These pleiotropic effects highlighted above position aldosterone synthase inhibitors as promising therapeutic agents for resistant hypertension, chronic kidney disease and heart failure. (Created in BioRender. Pradhan, A. (2026) https://BioRender.com/2dl87s5).

The regulation of CYP11B2 expression is primarily controlled by angiotensin II via AT1 receptor-mediated calcium–calmodulin signalling, as well as by extracellular potassium through membrane depolarisation and activation of T-type calcium channels. ACTH also contributes to regulation, although to a lesser extent [33,34]. Additionally, genetic variation within the CYP11B2 promoter region, most notably the −344 C/T polymorphism (rs1799998), has been associated with differences in transcriptional activity, aldosterone-to-renin ratio, blood pressure, and cardiovascular risk [35] (Figure 1).

### 3.2. Pathophysiological Consequences of Aldosterone Excess

The clinical relevance of aldosterone extends well beyond its classical role in the regulation of renal sodium balance. Although its acute physiological effects are adaptively facilitating sodium reabsorption and intravascular volume expansion in states of true hypovolaemia, chronic and inappropriate aldosterone excess, whether absolute (as observed in primary aldosteronism) or relative (as in heart failure, CKD, and resistant hypertension with elevated aldosterone-to-renin ratios), contributes to a broad spectrum of maladaptive processes. These collectively represent a major driver of cardiovascular and renal morbidity [36,37]. Within the distal nephron, aldosterone exerts its canonical actions by binding to intracellular mineralocorticoid receptors (MR) in principal cells of the distal convoluted tubule and cortical collecting duct. This interaction initiates genomic signalling pathways that increase the expression and apical membrane insertion of epithelial sodium channels (ENaC), along with upregulation of the basolateral Na^+^/K^+^-ATPase [38]. The resulting enhancement of electrogenic sodium reabsorption, coupled with obligate water retention, underlies the characteristic clinical phenotype of aldosterone excess, including volume expansion, hypertension, hypokalaemia, and metabolic alkalosis [39]. In primary aldosteronism, these mechanisms explain the discordance between suppressed plasma renin activity and the severity of hypertension, as well as the increased incidence of cardiovascular events such as stroke, atrial fibrillation, and myocardial infarction compared with essential hypertension, even when blood pressure levels are similar [40,41].

In addition to its renal effects, aldosterone exerts direct pathological actions in non-epithelial tissues through both MR-dependent and MR-independent pathways. In the myocardium, aldosterone promotes activation of pro-fibrotic signalling cascades, including transforming growth factor-beta (TGF-β), connective tissue growth factor (CTGF), and galectin-3. These pathways contribute to interstitial and perivascular fibrosis, impaired diastolic function, and increased susceptibility to ventricular arrhythmias [42,43]. Clinical evidence supporting the pathogenic role of aldosterone in cardiac remodelling is provided by landmark trials such as RALES and EPHESUS, which demonstrated significant mortality benefits with mineralocorticoid receptor antagonism in patients with heart failure with reduced ejection fraction [9,44].

In the vasculature, aldosterone contributes to endothelial dysfunction and adverse vascular remodelling. It impairs endothelium-dependent vasodilatation, enhances oxidative stress through activation of NADPH oxidase, promotes vascular smooth muscle cell hypertrophy, and increases arterial stiffness. Importantly, these effects contribute to cardiovascular risk independently of blood pressure elevation, highlighting the non-haemodynamic consequences of aldosterone excess [45].

Within the kidney, sustained aldosterone exposure has been implicated in direct structural injury [46]. Activation of MR in podocytes contributes to albuminuria and glomerulosclerosis, while effects on tubular epithelial cells promote transforming growth factor-beta-mediated epithelial-to-mesenchymal transition and tubulo-interstitial fibrosis processes increasingly recognised as central to CKD progression [47]. Epidemiological data in patients with primary aldosteronism demonstrate higher rates of CKD and progression to end-stage renal disease compared with individuals with essential hypertension, even after adjustment for blood pressure and comorbidities such as diabetes. These findings support the concept that aldosterone exerts direct nephrotoxic effects beyond its haemodynamic influence [48].

Collectively, the multi-organ pathological effects of aldosterone provide a strong biological rationale for therapeutic strategies aimed at suppressing aldosterone synthesis itself, rather than solely antagonising its receptor or modulating upstream components of the RAAS.

### 3.3. Limitations of Existing RAAS-Targeted Therapies

Despite the central role of the RAAS in cardiovascular and renal pathophysiology, currently available pharmacological strategies targeting this axis are associated with important mechanistic and clinical limitations. These shortcomings have driven the development of ASIs as a novel and complementary therapeutic class [49,50]. A critical evaluation of these limitations is essential to appropriately position ASIs within the contemporary therapeutic landscape.

Angiotensin-converting enzyme (ACE) inhibitors and angiotensin receptor blockers (ARBs) are the most widely utilised RAAS-modulating agents and have consistently demonstrated substantial clinical benefits in hypertension, heart failure, and diabetic nephropathy in landmark outcome trials [51]. However, both classes are limited by the well-recognised phenomenon of aldosterone breakthrough, characterised by the re-elevation of plasma aldosterone concentrations to baseline or suprabasal levels during chronic therapy, occurring in approximately 40% of patients receiving long-term treatment [52,53]. Mechanistically, this phenomenon is attributed to persistent aldosterone synthesis via angiotensin II-independent pathways, including potassium-mediated stimulation of the zona glomerulosa [secondary to RAAS blockade], adrenocorticotropic hormone (ACTH)-mediated adrenal activation, and intra-adrenal autocrine/paracrine signalling that maintains CYP11B2 expression independently of circulating angiotensin II [54]. Clinically, aldosterone breakthrough may attenuate the cardiorenal protective effects of ACE inhibitors and ARBs, thereby contributing to residual cardiovascular risk despite therapy [55]. Although ”Sartans” represent the prototype ARBs, recent evidence suggests that many novel large peptide and small non-peptide molecules and AT1 receptor blockers are in the pipeline [56].

Moreover, these agents fail to suppress aldosterone production in conditions such as primary aldosteronism, where autonomous overexpression of CYP11B2 renders aldosterone synthesis independent of angiotensin II regulation [57].

Mineralocorticoid receptor antagonists (MRAs), including the steroidal agents spironolactone and eplerenone, as well as the non-steroidal agent finerenone, mitigate aldosterone-mediated end-organ damage by competitively inhibiting the mineralocorticoid receptor and thereby blocking downstream genomic effects [58]. MRAs have demonstrated robust cardiovascular and renal benefits across major clinical trials such as RALES, EPHESUS, EMPHASIS-HF, FIDELIO-DKD, and FIGARO-DKD, and are firmly established in current guidelines for the management of heart failure and chronic kidney disease [59,60]. Nevertheless, their use is constrained by several clinically relevant limitations. Steroidal MRAs, particularly spironolactone, exhibit significant off-target activity at androgen and progesterone receptors, leading to dose-dependent adverse effects such as gynaecomastia, sexual dysfunction, and menstrual irregularities, which may impair tolerability and adherence [61]. Eplerenone, although more selective, has comparatively lower affinity for the mineralocorticoid receptor, necessitating higher dosing to achieve similar efficacy and demonstrating less pronounced antihypertensive effects in resistant hypertension [62]. Finerenone, a newer non-steroidal MRA, offers improved cardiorenal tissue selectivity and avoids sex hormone-related adverse effects; however, it remains associated with a class effect risk of hyperkalaemia and does not suppress circulating aldosterone levels. Indeed, MR blockade may lead to a compensatory rise in aldosterone concentrations due to the disruption of negative feedback mechanisms, potentially allowing persistence of non-genomic and receptor-independent aldosterone effects [63,64].

Importantly, none of the currently available RAAS-directed therapies including ACE inhibitors, ARBs, and MRAs directly inhibit aldosterone synthesis. This represents a key pharmacological limitation, as circulating aldosterone levels may remain elevated or even increase during therapy, and its non-genomic pro-inflammatory and vascular effects may not be fully mitigated by receptor blockade alone [65]. In contrast, direct inhibition of CYP11B2 targets aldosterone production at its enzymatic source, reducing circulating levels and potentially suppressing both genomic and non-genomic pathways of aldosterone-mediated tissue injury. This mechanistic advantage underpins the rationale for the development of ASIs as an emerging therapeutic strategy within the RAAS framework [66].

## 4. CYP11B2 vs. CYP11B1: The Selectivity Challenge That Defined ASI Development

The development of clinically viable ASIs has been fundamentally influenced and, for nearly two decades, limited by a critical pharmacological challenge: achieving adequate selectivity for CYP11B2 over its closely related paralogue, CYP11B1 (steroid 11β-hydroxylase) [67,68]. This issue extends beyond a conventional drug development obstacle and reflects a deeper mechanistic constraint rooted in the evolutionary conservation and structural similarity of the CYP11B enzyme family. This challenge ultimately dictated the failure of first-generation ASIs and informed the structural innovations underpinning second-generation compounds.

CYP11B1 and CYP11B2 are mitochondrial cytochrome P450 enzymes encoded by adjacent genes on chromosome 8q22, arising from a gene duplication event approximately 40 million years ago [69]. Despite mediating distinct physiological processes CYP11B1 catalyses the 11β-hydroxylation of 11-deoxycortisol to cortisol in the zona fasciculata, whereas CYP11B2 facilitates the multistep conversion of deoxycorticosterone (DOC) to aldosterone within the zona glomerulosa the two enzymes exhibit approximately 93% amino acid sequence homology, with highly conserved active site architecture [70]. Structural analyses, including X-ray crystallography, have demonstrated that the limited amino acid differences between CYP11B1 and CYP11B2 are predominantly located in regions governing substrate access and orientation rather than within the catalytic haem-binding core. This high degree of structural overlap significantly complicates the rational design of selective inhibitors [71,72].

Insufficient selectivity for CYP11B2 has important and clinically consequential implications. Inhibition of CYP11B1 reduces cortisol synthesis, leading to compensatory activation of the hypothalamic–pituitary–adrenal axis and increased secretion of adrenocorticotropic hormone (ACTH) due to loss of negative feedback [73]. Elevated ACTH enhances steroidogenic flux upstream of both CYP11B1 and CYP11B2, resulting in accumulation of DOC, which cannot be efficiently metabolised when CYP11B1 is inhibited [74]. At supraphysiological concentrations, DOC exerts mineralocorticoid activity, activating the mineralocorticoid receptor with approximately one-third the potency of aldosterone and thereby paradoxically exacerbating sodium retention and hypertension [75]. This mechanism is exemplified by clinical observations with LCI699 in hypertension studies, where marked elevations in plasma DOC (approximately 700–1400% above baseline) were observed despite effective suppression of aldosterone, ultimately attenuating antihypertensive efficacy [76]. Furthermore, CYP11B1 inhibition may impair cortisol production sufficiently to pose a risk of relative adrenal insufficiency, particularly under conditions of physiological stress, with potential progression to adrenal crisis in vulnerable settings such as surgery or critical illness [77].

Efforts to overcome this selectivity challenge have leveraged advances in structural biology, including homology modelling, molecular docking, and structure-based drug design following the elucidation of CYP11B1 and CYP11B2 crystal structures [78,79]. Subtle but functionally important differences in active site residues such as Leu301 in CYP11B1 versus Val301 in CYP11B2, and Ala320 in CYP11B1 versus Gly320 in CYP11B2 have been identified as key determinants influencing ligand binding orientation and selectivity [80]. Second-generation ASIs have been rationally designed to exploit these differences through distinct chemical scaffolds, including tetrahydroisoquinoline derivatives (e.g., baxdrostat), imidazole-based heterocycles (e.g., lorundrostat), and enantiomerically pure pyridine analogues (e.g., dexfadrostat). These compounds achieve selective binding conformations that are sterically or energetically unfavourable within the CYP11B1 active site, resulting in selectivity ratios ranging from approximately 30:1 to greater than 100:1 [81,82]. Importantly, this enhanced selectivity has been consistently validated in clinical development programmes, as evidenced by the absence of significant cortisol suppression or DOC accumulation. This pharmacological refinement represents the key advance distinguishing second-generation ASIs and underlies their successful progression through clinical development, in contrast to earlier agents (Figure 1).

### 4.1. First-Generation Aldosterone Synthase Inhibitors: Proof of Concept and Mechanistic Limitations

The first-generation ASIs that are FAD286, LCI699 (Osilodrostat), and LY3045697 collectively established the pharmacological proof of concept that CYP11B2 is an inhibitable, clinically relevant target whose blockade produces measurable reductions in aldosterone synthesis, blood pressure, and potassium wasting in humans. However, each of these agents was ultimately limited by mechanistic liabilities rooted in insufficient CYP11B2/CYP11B1 selectivity or inadequate potency, preventing clinical advancement in the cardiovascular and renal indications for which they were originally intended. Their development nonetheless generated critical insights regarding the structural requirements for ASI selectivity that directly informed the design of second-generation compounds [65,83].

FAD286, the dextro-enantiomer of the aromatase inhibitor fadrozole, was the first compound characterised as a selective CYP11B2 inhibitor in preclinical models, demonstrating dose-dependent suppression of plasma aldosterone and attenuation of hypertension-related cardiac fibrosis and hypertrophy in spontaneously hypertensive rats and transgenic renin-overexpressing murine models [84,85]. However, FAD286 also exhibited inhibition of CYP11B1 at supratherapeutic concentrations, and preclinical studies demonstrated paradoxical intestinal inflammatory effects when combined with spironolactone, partly attributed to upregulation of colonic aldosterone production through compensatory adrenal stimulation [86]. FAD286 was never advanced to formal human Phase I studies, serving primarily as a pharmacological tool compound and structural template for subsequent ASI development [87].

LCI699, subsequently developed and licenced as osilodrostat (Isturisa^®^) by Novartis and later Recordati, represented the first ASI to demonstrate clinical proof of concept in human studies. In an early Phase II study by Azizi and colleagues, LCI699 produced dose-dependent suppression of plasma aldosterone by up to 80% at doses of 0.5 mg twice daily and significant reductions in systolic blood pressure in patients with primary aldosteronism and low-renin essential hypertension [75]. Potassium normalisation was observed in hypokalaemic patients, validating the mechanism of aldosterone suppression in vivo. However, even at low doses, LCI699 produced marked accumulation of DOC rising by 710% to 1427% above baseline across dose cohorts and significant suppression of the cortisol response to ACTH stimulation, confirming clinically relevant CYP11B1 off-target activity [73]. Subsequent Phase II studies in resistant hypertension confirmed these findings and demonstrated that the mineralocorticoid activity of accumulated DOC substantially attenuated the blood pressure-lowering effect of aldosterone suppression, rendering the net antihypertensive effect of LCI699 clinically insufficient to justify progression in hypertension [75]. Recognising that CYP11B1 inhibition and cortisol suppression were in fact pharmacologically desirable in the context of Cushing’s disease, where autonomous cortisol excess is the therapeutic target, Novartis pivoted the clinical programme to hypercortisolism, culminating in the successful Phase III LINC 3 and LINC 4 trials and FDA approval of osilodrostat (Isturisa^®^) for Cushing’s disease in March 2020 [88]. Real-world data from a TriNetX database analysis reported at the American Heart Association Scientific Sessions 2025 noted that osilodrostat demonstrated blood pressure control in 60% of a small cohort of hypertensive patients at 52 weeks, though these findings require prospective validation and do not address the DOC accumulation concern at hypertension-relevant doses [89].

LY3045697, developed by Eli Lilly, was designed to address the CYP11B1 off-target problem of LCI699 through a novel non-imidazole structural scaffold that demonstrated superior CYP11B2/CYP11B1 selectivity in vitro [90]. Two Phase II randomised controlled trials confirmed improved selectivity, with minimal cortisol suppression at therapeutic doses. However, the compound exhibited insufficient potency at doses that preserved CYP11B1 selectivity, necessitating dose escalation for adequate aldosterone suppression, at which point selectivity was compromised and cortisol-axis effects re-emerged [90]. This potency-selectivity trade-off, inherent to a suboptimal structural scaffold, rendered LY3045697 clinically unviable, and its development was discontinued. The compound nonetheless validated the concept that CYP11B2/CYP11B1 selectivity was achievable in humans and that a sufficiently selective agent could suppress aldosterone without DOC accumulation—a conclusion that guided the structural chemistry programmes that produced the second-generation ASIs [91].

### 4.2. Second-Generation Aldosterone Synthase Inhibitors: Structural Innovation and Clinical Advancement

The development of second-generation ASIs was driven by advances in structural biology, particularly the elucidation of the first crystal structures of human CYP11B1 and CYP11B2, alongside iterative medicinal chemistry efforts aimed at overcoming the limitations of first-generation compounds [92,93]. Collectively, these newer agents share three defining pharmacological characteristics that distinguish them from earlier ASIs: marked selectivity for CYP11B2 over CYP11B1 [typically ranging from 30:1 to >100:1], preservation of cortisol biosynthesis across therapeutic dose ranges, and suitability for once-daily oral administration owing to favourable pharmacokinetic profiles with half-lives generally between 15 and 30 h [94]. Clinical development programmes have consistently demonstrated that selective CYP11B2 inhibition results in sustained aldosterone suppression, clinically meaningful reductions in blood pressure, and particularly in agents studied in CKD-significant reductions in albuminuria. These findings position second-generation ASIs as a promising addition to the contemporary cardiorenal therapeutic armamentarium.

#### 4.2.1. Baxdrostat

Baxdrostat (CIN-107; AstraZeneca) is currently the most clinically advanced second-generation ASI and the first agent in this class to demonstrate positive findings in a Phase III cardiovascular outcomes programme. Structurally, baxdrostat incorporates a tetrahydroisoquinoline scaffold that achieves a CYP11B2/CYP11B1 selectivity ratio exceeding 100:1, the highest reported among clinical-stage ASIs. This selectivity is mediated through a binding configuration that is sterically favoured within the CYP11B2 active site but disfavoured within CYP11B1 [81]. Baxdrostat is administered once daily and has a terminal half-life of approximately 29–30 h, allowing sustained 24 h aldosterone suppression with predictable pharmacokinetics that are minimally influenced by renal impairment at doses up to 2 mg [81].

Phase I studies demonstrated dose-proportional pharmacokinetics and confirmed the absence of clinically relevant CYP11B1 inhibition at doses up to 10 mg in healthy volunteers, with preservation of both basal and ACTH-stimulated cortisol levels across all dose cohorts [94]. The Phase II **BrigHTN** trial randomised 248 patients with treatment-resistant hypertension, defined as uncontrolled blood pressure despite treatment, with at least three antihypertensive agents, including a diuretic to baxdrostat 0.5 mg, 1 mg, or 2 mg once daily, versus placebo [93]. Baxdrostat 1 mg and 2 mg achieved significant dose-dependent reductions in seated systolic blood pressure (SBP) compared with placebo, with the 2 mg dose producing an approximately 11 mmHg placebo-adjusted SBP reduction at 12 weeks. Plasma aldosterone concentrations were suppressed across all active treatment groups, while cortisol concentrations and ACTH-stimulated cortisol responses remained preserved, confirming effective CYP11B2 selectivity in humans. Hyperkalaemia, defined as serum potassium >5.5 mmol/L, occurred infrequently and demonstrated a dose-related pattern; however, no severe hyperkalaemic events requiring emergency intervention were reported.

The parallel **HALO** trial, which evaluated baxdrostat in patients with uncontrolled hypertension receiving fewer than three antihypertensive medications, failed to meet its primary endpoint of SBP reduction at 12 weeks compared with placebo. This outcome was attributed in part to greater variability in medication adherence and a stronger placebo response in the non-resistant hypertension population, despite effective aldosterone suppression across all active-dose groups [95].

Baxdrostat subsequently demonstrated proof-of-concept efficacy in primary aldosteronism in the Phase IIa **SPARK** trial, published in 2025. Treatment with baxdrostat 0.5–2 mg resulted in resolution or substantial improvement of hypertension, hypokalaemia, and elevated aldosterone-to-renin ratio in patients with confirmed primary aldosteronism, irrespective of whether the underlying pathology was unilateral adenoma or bilateral adrenal hyperplasia [96]. These findings provided preliminary evidence supporting medical management of primary aldosteronism as a potential alternative to adrenalectomy in selected patients.

The pivotal Phase III **BaxHTN** trial enrolled 796 patients with resistant hypertension across multiple countries and met its primary endpoint, demonstrating statistically and clinically significant reductions in seated SBP at 12 weeks with both the 1 mg and 2 mg doses. Placebo-adjusted SBP reductions were approximately 10 mmHg (95% CI: −12.6 to −7.0; *p* < 0.01) [92]. All prespecified secondary endpoints, including reductions in diastolic blood pressure and achievement of SBP < 130 mmHg, were also met. The safety profile remained favourable, with manageable dose-related hyperkalaemia representing the principal adverse event and no evidence of adrenal insufficiency or cortisol suppression.

In parallel, the Phase III **Bax24** trial evaluated 24 h ambulatory systolic blood pressure after 12 weeks of treatment as the primary endpoint in 854 patients with diagnosed resistant hypertension [97]. At 12 weeks, baxdrostat therapy produced a significantly lower 24 h ambulatory SBP (least mean square) by 14.4 mg (95% CI: −17.2 mm Hg to −10.8 mm Hg; *p* < 0.0001) compared to the placebo arm. These reported positive results further support the durability of baxdrostat’s antihypertensive effect throughout the circadian blood pressure cycle. Following the regulatory submission to the FDA, the drug has been approved by the agency for management of uncontrolled hypertension in May 2026 [98]. Meanwhile, additional Phase III programmes, including BaxAsia (Asia-Pacific hypertension), FigHTN-CKD (Hypertension with CKD), and a heart failure prevention study, are ongoing to further elaborate the role of the molecule in the spectrum of cardio–kidney–metabolic syndrome.

#### 4.2.2. Lorundrostat

Lorundrostat (MLS-101; Mineralys Therapeutics) is the second most advanced second-generation ASI and the first agent within this class to demonstrate clinically significant reductions in albuminuria in patients with CKD, thereby extending the therapeutic potential of ASIs beyond blood pressure reduction toward cardiorenal protection. Lorundrostat possesses a structurally distinct scaffold from baxdrostat while maintaining high selectivity for CYP11B2 over CYP11B1, with preservation of cortisol biosynthesis consistently demonstrated across clinical studies [82].

The Phase II **TARGET-HTN** trial, published in 2023, evaluated lorundrostat at doses of 50 mg, 100 mg, and 200 mg once daily in 163 patients with uncontrolled hypertension receiving background RAAS blockade [82]. Lorundrostat produced dose-dependent and clinically meaningful reductions in automated office SBP, exceeding 14 mmHg in the highest-dose cohort. Hyperkalaemia represented the principal adverse event but was generally mild and transient, with no reported cases of severe hyperkalaemia or adrenal insufficiency.

Subsequently, the Phase II **Advance-HTN** trial demonstrated significant reductions in 24 h ambulatory SBP at 12 weeks among patients with uncontrolled and resistant hypertension, thereby supporting the durability of lorundrostat’s antihypertensive efficacy over a full dosing interval [99].

The pivotal Phase III **Launch-HTN** trial enrolled 1083 patients across 13 countries and was published in 2025 [100]. The study met its primary endpoint, demonstrating a statistically significant placebo-adjusted reduction in automated office SBP of approximately 9.1 mmHg at week 6 (95% CI: −13.3 to −4.9 mm Hg; *p* < 0.001), with consistent benefit observed in both uncontrolled and resistant hypertension subgroups. A subsequent meta-analysis pooling data from the three lorundrostat randomised controlled trials reported a pooled relative risk of hyperkalaemia of 3.22 (95% CI: 2.01 to 5.15; *p* < 0.001) compared with placebo, reinforcing hyperkalaemia as the principal class-wide safety signal while noting that rates of serious adverse events remained low and comparable between treatment groups [101].

Particularly noteworthy were findings from the Phase II **Explore-CKD** trial, presented at the American Society of Nephrology (ASN) in November 2025. In patients with albuminuric CKD and hypertension receiving background ACE inhibitors or ARB therapy, lorundrostat achieved a placebo-adjusted office SBP reduction of 9.3 mmHg and, importantly, a statistically significant 25.6% placebo-adjusted reduction in urine albumin-to-creatinine ratio (UACR; *p* = 0.0015) [102]. This represented the first prospective demonstration of ASI-mediated albuminuria reduction and suggested a potential reno-protective mechanism extending beyond blood pressure control. These findings have positioned lorundrostat as a promising candidate for future CKD outcome trials. Mineralys Therapeutics has announced plans for FDA submission based on the Launch-HTN data, while the EXPLORE-OSA trial (NCT06785454) evaluating lorundrostat in obstructive sleep apnoea-associated hypertension is ongoing, with results anticipated in 2026.

#### 4.2.3. Vicadrostat (BI 690517)

Vicadrostat [BI 690517; Boehringer Ingelheim in collaboration with Eli Lilly] is the ASI most strategically positioned within the cardiorenal outcome landscape, with the most advanced development programme in CKD and the only agent in this class currently undergoing large-scale Phase III cardiovascular and renal outcomes evaluation. In contrast to baxdrostat and lorundrostat, which were initially developed primarily for hypertension, vicadrostat has been specifically designed as an organ-protective therapy targeting aldosterone-mediated renal and cardiac injury in CKD and heart failure [103].

Four Phase I studies conducted in European and Asian (Chinese and Japanese) healthy volunteer populations demonstrated dose-proportional pharmacokinetics, favourable tolerability, and complete preservation of CYP11B1 function, with no reduction in basal or ACTH-stimulated cortisol levels across all studied doses [104].

The pivotal Phase II CKD trial (NCT05182840) enrolled 586 patients with albuminuric CKD and evaluated vicadrostat 5 mg or 10 mg once daily with and without background empagliflozin in a 2 × 2 factorial design [105]. Vicadrostat 10 mg achieved an approximately 40% reduction in UACR compared with placebo, with benefit observed irrespective of concomitant empagliflozin therapy. Notably, combined treatment with vicadrostat and empagliflozin was associated with attenuation of the hyperkalaemia signal observed with vicadrostat monotherapy. This finding is pharmacodynamically plausible, reflecting SGLT2 inhibitor-mediated natriuresis and mild kaliuresis counterbalancing the potassium-retaining effects of aldosterone suppression, and provides a clinically relevant strategy for mitigating hyperkalaemia risk in CKD populations [105].

Further confirmation of CYP11B2 selectivity was provided through a post hoc LC-MS/MS steroid metabolomics analysis presented at ASN Kidney Week 2025, which demonstrated complete preservation of cortisol and related steroid metabolites across all vicadrostat dose groups throughout the study period [106].

Three large-scale Phase III outcome trials are currently ongoing. The EASi-KIDNEY trial (NCT06531824)is an event-driven, double-blind, placebo-controlled study evaluating vicadrostat in patients with albuminuric CKD, with a composite primary endpoint including kidney failure, sustained ≥40% decline in estimated glomerular filtration rate (eGFR), cardiovascular death, or first hospitalisation for heart failure [103]. This represents the first ASI outcome trial to incorporate kidney failure as a major primary endpoint component.

In parallel, the EASi-HF Preserved trial (NCT06424288) and EASi-HF Reduced trial (NCT06935370) are evaluating vicadrostat in heart failure with preserved and reduced ejection fraction, respectively, with co-primary endpoints of cardiovascular death and heart failure hospitalisation across approximately 6000 patients per study. Collectively, these programmes represent the most extensive clinical evaluation undertaken for any ASI and may establish vicadrostat as the first agent in this class with proven cardiovascular and renal outcome benefits.

#### 4.2.4. Dexfadrostat Phosphate

Dexfadrostat phosphate (Idorsia/Recordati) is the most recently characterised second-generation ASI. The compound represents the enantiopure dextrorotatory isomer of fadrozole, generated through proprietary asymmetric crystallisation techniques achieving 99.9% enantiomeric excess [107]. Similar to other second-generation ASIs, dexfadrostat demonstrates high selectivity for CYP11B2 over CYP11B1, although the absolute selectivity ratio is lower than that reported for baxdrostat. Nevertheless, therapeutic dosing has shown minimal effects on cortisol biosynthesis.

The Phase I dose-escalation study, published in 2023, evaluated single ascending doses ranging from 1 mg to 40 mg in 40 healthy male volunteers [108]. Dexfadrostat produced dose-dependent suppression of plasma aldosterone and ARR, with minimal impact on serum cortisol concentrations at doses up to 16 mg.

Subsequently, the Phase II primary aldosteronism trial (NCT04007406; EudraCT 2019-000919-85), published in 2024, randomised approximately 90 patients with confirmed primary aldosteronism to dexfadrostat 4 mg, 8 mg, or 12 mg once daily versus placebo for 8 weeks [109]. All active-dose groups demonstrated statistically significant reductions in ARR and SBP relative to placebo, alongside correction of hypokalaemia in affected patients. The treatment was generally well tolerated, with headache and dizziness representing the most commonly reported adverse events. No cases of adrenal insufficiency were observed. However, dose-dependent increases in corticosterone concentrations were noted at the 12 mg dose, suggesting residual upstream steroidogenic flux despite the absence of overt CYP11B1 inhibition within the therapeutic range [109].

### 4.3. Class-Wide Adverse Effect Profile: Hyperkalaemia and Mitigation Strategies

Hyperkalaemia represents the most clinically significant and consistently observed adverse effect across all ASI, irrespective of generation or structural class, and constitutes the principal safety challenge that will need to be managed at the population level if ASIs achieve widespread clinical adoption [110]. The mechanistic basis for ASI-associated hyperkalaemia is directly and inextricably linked to the pharmacological action of the drug class: aldosterone drives renal potassium excretion through MR-mediated upregulation of luminal potassium channels [ROMK] in the collecting duct, and suppression of aldosterone biosynthesis by CYP11B2 inhibition reduces this kaliuretic drive, resulting in potassium retention proportional to the degree of aldosterone suppression [111].

Pooled data from randomised controlled trials of lorundrostat demonstrate a relative risk of hyperkalaemia [serum potassium > 5.0–5.5 mmol/L] of 7.93 compared with placebo, while published Phase II and III data for baxdrostat report dose-related hyperkalaemia rates of 3–8% at 1 mg and 2 mg doses respectively in resistant hypertension populations [92,93,112]. Importantly, the great majority of hyperkalaemia events observed across ASI clinical trials have been mild to moderate (serum K^+^ 5.0–5.9 mmol/L) and clinically manageable through dose reduction or dietary potassium modification, and have not been associated with serious adverse cardiac outcomes. No cases of life-threatening hyperkalaemia (serum K^+^ > 6.5 mmol/L) or hyperkalaemia-related mortality have been reported in Phase II or III trials to date [92,97]. Rates of serious adverse events attributable to hyperkalaemia across the baxdrostat BrigHTN trial were limited to only two of 248 participants, and no cases required emergency intervention or premature trial discontinuation in the Launch-HTN Phase III trial [93,99].

The prescribing information for baxdrostat (Baxfendy^R^, AstraZeneca Inc.) recommends evaluation of serum potassium and management of hyperkalaemia prior to initiation of therapy. Periodic monitoring of serum potassium is mandated while patients with pre-existing risk factors for hyperkalaemia, including advanced CKD (eGFR below 45 mL/min/1.73 m^2^), diabetes mellitus, concomitant MRA or potassium-sparing diuretic use, and high dietary potassium intake, will benefit from more frequent monitoring [113]. However, the FDA label does not specify a universal potassium-based dose-reduction algorithm or a fixed interval for repeat testing after treatment initiation or dose escalation; therefore, standardised, prospectively validated monitoring and management pathways, including the potential role of potassium binders and SGLT2 inhibitors, remain important areas for future research, particularly in patients with CKD and those receiving concomitant RAAS-modifying therapy. The combination of vicadrostat with empagliflozin in the Phase II CKD trial demonstrated a clinically and pharmacodynamically coherent reduction in hyperkalaemia risk, reflecting the natriuretic and mild kaliuretic effects of SGLT2 inhibition counterbalancing aldosterone-suppression-mediated potassium retention [105]. This combination strategy has been incorporated into the EASi-KIDNEY Phase III design and may represent a broadly applicable approach to hyperkalaemia mitigation across the ASI class, particularly in the CKD population where both SGLT2 inhibitors and RAAS blockade are already guideline-recommended [114]. Potassium binders such as patiromer and sodium zirconium cyclosilicate represent additional pharmacological tools for hyperkalaemia management that could expand the eligible patient population for ASI therapy [115,116].

Beyond hyperkalaemia, mild hyponatraemia has been reported at low incidence across multiple ASI trials, reflecting the volume-regulatory consequences of aldosterone suppression, but has not been clinically significant in any published trial to date [93,100]. Transient and reversible reductions in eGFR of approximately 2–4 mL/min/1.73 m^2^ have been observed at treatment initiation with lorundrostat in CKD populations, consistent with the haemodynamic consequences of reduced aldosterone-mediated glomerular hyperfiltration, analogous to the early eGFR dip observed with ACE inhibitors, ARNI and SGLT-2 inhibitors [117]. Postural hypotension has been reported rarely and appears to be more common in patients with volume depletion or concomitant loop diuretic use. Importantly, no cases of adrenal insufficiency, cortisol suppression, or clinically significant elevation of DOC have been reported in any Phase II or Phase III trial of any second-generation ASI, confirming that the CYP11B2 selectivity achieved structurally has translated fully into clinical practice [81,82].

### 4.4. Comparative Efficacy: ASIs Versus Mineralocorticoid Receptor Antagonists

The emergence of second-generation ASIs inevitably invites direct comparison with the established MRA class, particularly finerenone, which has itself recently demonstrated cardiorenal outcome benefits across two large Phase III trials in CKD associated with type 2 diabetes [60,118]. A nuanced pharmacological and clinical comparison between ASIs and MRAs is essential for positioning ASIs within the existing therapeutic hierarchy and for identifying patient populations and clinical contexts in which one approach may be preferred over the other.

At the mechanistic level, ASIs and MRAs share the common goal of reducing aldosterone-mediated end-organ injury but achieve this through fundamentally different and potentially complementary mechanisms. MRAs competitively block the MR at the receptor level, preventing aldosterone from exerting its downstream genomic effects but leaving circulating aldosterone concentrations unaffected or even elevated through loss of feedback suppression [58]. ASIs, by contrast, reduce circulating aldosterone concentrations by directly inhibiting its synthesis, thereby extinguishing both MR-mediated genomic effects and the growing body of non-genomic, MR-independent actions of aldosterone on vascular inflammation, oxidative stress, and cardiac fibrosis [119]. This distinction is particularly relevant in the context of primary aldosteronism, where chronically elevated circulating aldosterone rather than heightened tissue MR sensitivity is the primary pathological driver, and where source-targeted aldosterone synthesis inhibition may be mechanistically superior to receptor-level blockade [39].

In terms of blood pressure reduction in resistant hypertension, arguably the most directly comparable clinical endpoint, published Phase III data suggest broadly similar absolute magnitudes of SBP reduction for ASIs (approximately 9–10 mmHg placebo-adjusted for lorundrostat and baxdrostat respectively in Phase III) and spironolactone (approximately 8–15 mmHg in resistant hypertension trials including PATHWAY-2) [92,100,120]. However, direct head-to-head comparative trials between ASIs and MRAs in resistant hypertension have not been conducted, and indirect comparisons are confounded by differences in patient populations, background therapy, and endpoint definitions across trials. A published Bayesian network meta-analysis estimated a pooled placebo-adjusted SBP reduction of −11.1 mmHg for baxdrostat and −9.1 mmHg for lorundrostat in resistant hypertension, which appears comparable to the estimates for spironolactone from the PATHWAY-2 trial (~8.7 mmHg); however, this comparison does not account for the substantially superior tolerability and absence of sex hormone side effects with ASIs [121].

The tolerability advantage of ASIs over steroidal MRAs is clinically meaningful and likely to have important implications for treatment adherence and long-term outcomes. Spironolactone-associated gynaecomastia, sexual dysfunction, and menstrual irregularities represent a major cause of discontinuation, particularly in younger male patients, and are absent with ASIs by virtue of their highly specific enzymatic mechanism of action [61]. Finerenone, while avoiding sex hormone receptor side effects, shares the class-wide hyperkalaemia risk of MRAs and has not demonstrated superiority over spironolactone in direct blood pressure reduction [63]. The hyperkalaemia risk profile of ASIs and MRAs appears broadly comparable, though the precise mechanistic drivers differing in MRA-associatedhyperkalaemia results from MR blockades in the collecting duct reducing potassium excretion, while ASI-associated hyperkalaemia results from aldosterone suppression reducing the same kaliuretic drive [122].

The unresolved and clinically critical question is whether ASIs can demonstrate hard cardiovascular and renal outcome benefit reductions in MACE, kidney failure, and cardiovascular mortality comparable to or exceeding those already established for finerenone in the FIDELIO-DKD and FIGARO-DKD trials [116,123]. The ongoing EASi-KIDNEY, EASi-HF Preserved, and EASi-HF Reduced trials with vicadrostat will provide the first definitive outcome data for the ASI class and will directly address whether synthesis suppression offers organ-protective advantages over receptor blockades. Until these data are available, MRAs retain a stronger evidence base for outcome-driven prescribing decisions in CKD and heart failure, and ASIs should be considered alongside rather than as a replacement for established MRA therapy in these indications. Table 1 gives an overview of the various ASIs—highlighting their mechanisms of action, clinical development, safety findings, and major therapeutic outcomes in hypertension and cardiorenal disease.

## 5. Future Perspectives and Unresolved Questions

The second-generation aldosterone synthase inhibitors represent a major advance in RAAS pharmacology, particularly because selective CYP11B2 inhibition has progressed from proof-of-concept studies to late-stage clinical development and, for baxdrostat, regulatory approval for hypertension. Nevertheless, the class remains under evaluation for long-term cardiorenal outcomes and broader clinical positioning. Nevertheless, several important scientific and clinical questions remain unresolved and will shape the ultimate positioning and clinical impact of ASIs within the cardiorenal therapeutic landscape.

The most pressing unresolved question is whether ASI therapy translates into hard cardiovascular and renal outcome benefit reductions in myocardial infarction, stroke, kidney failure, and cardiovascular mortality comparable to the outcome benefits established for SGLT2 inhibitors, GLP-1 receptor agonists, and finerenone, which now occupy central positions in cardiorenal management guidelines. The ongoing EASi-KIDNEY, EASi-HF Preserved, and EASi-HF Reduced trials with vicadrostat will provide the first outcome data for the class but are not expected to report primary results until 2028–2030, leaving a prolonged period of clinical uncertainty during which ASIs (baxdrostat) have received regulatory approval from the USFDA for blood pressure indications without established outcome evidence [98,103]. Regulatory agencies and clinical guidelines committees will face the novel challenge of determining appropriate positioning for agents with robust blood pressure and surrogate endpoint benefits but without mature hard outcome data—a situation not dissimilar to the early post-approval period for SGLT2 inhibitors in hypertension.

A second critical unresolved question concerns the long-term adrenal safety of sustained CYP11B2 inhibition beyond the 12–24-week durations studied in existing Phase III trials. While all second-generation ASIs have demonstrated preservation of cortisol biosynthesis and the absence of adrenal insufficiency within trial periods, the long-term consequences of sustained reduction in circulating aldosterone, including potential compensatory adrenal hypertrophy, upregulation of CYP11B2 expression, and changes in adrenal zona glomerulosa morphology, have not been systematically evaluated in humans beyond 52 weeks [67]. Extended follow-up data from the EASi-KIDNEY and EASi-HF outcome trials, which will follow patients for median durations of three or more years, will be essential for characterising the long-term adrenal safety profile of the class.

The potential for combination therapy strategies, most notably ASI-plus-SGLT2 inhibitor, ASI-plus-MRA, and ASI-plus-direct renin inhibitor (DRI), represents a frontier of pharmacological investigation that may amplify organ-protective benefits while managing tolerability trade-offs. The first-generation DRI Aliskiren was withdrawn from the market after the seminal ALTITUDE trial failed to demonstrate any meaningful benefit, producing signals of harm leading to premature termination instead [124]. But a recent study has validated a novel lipophilic cyclo-octanoyl direct renin inhibitor scaffold via computational strategies in an in silico model to overcome the various pharmacokinetic shortcomings of Aliskiren [125]. The vicadrostat-plus-empagliflozin combination has demonstrated hyperkalaemia mitigation in Phase II CKD data, and the EASi-KIDNEY trial design incorporates background SGLT2 inhibitor therapy as a standard-of-care element, effectively testing this combination in a Phase III outcome setting [105]. Whether the addition of an MRA to an ASI provides additive MR blockade benefits by targeting both synthesis reduction and receptor blockade simultaneously or simply compounds the hyperkalaemia risk without additional clinical benefit is a pharmacodynamic question that requires dedicated investigation [63].

Renal denervation therapy now offers an additional line therapy for resistant hypertension with a mean daytime ambulatory SBP of roughly 6 mm Hg (95% CI, −8.1 to −3.8 mm Hg; *p*  <  0.001) [126]. Although this is comparable to the SBP reduction achieved, ASI therapy head-to-head trials are lacking. Although the ASIs have a minimal renal excretion, baxdrostat has been studied in eGFR up to 25 mL/min/m^2^, while lorundrostat has been studied in patients with eGFR > 45 mL/min/m^2^ [99,127].

Pregnancy remains a major evidence gap: the baxdrostat prescribing information reports no available human data to assess the risks of major congenital malformations, miscarriage, or adverse maternal or foetal outcomes, while animal studies demonstrated embryo-foetal toxicity at exposures more than 29-fold the human exposure at the clinical 2 mg dose; prospective pregnancy surveillance and dedicated pharmacovigilance will therefore be important before the role of ASIs during pregnancy can be defined [113]. Data in hepatic impairment also remain limited; for baxdrostat, exposure was similar in patients with moderate hepatic impairment (Child–Pugh B), whereas severe hepatic impairment has not been studied [113]. Drug–drug interactions warrant continued characterisation because baxdrostat is primarily metabolised by CYP3A4; although coadministration with itraconazole, a strong CYP3A/P-glycoprotein inhibitor, did not produce a clinically significant change in exposure, strong or moderate CYP3A inducers may reduce baxdrostat concentrations and antihypertensive efficacy. Concomitant drugs that increase serum potassium may further increase the risk of hyperkalaemia and warrant more frequent monitoring. Accordingly, current prescribing information recommends assessment of serum potassium and sodium before treatment initiation and, periodically thereafter, with more frequent monitoring in patients at increased risk, including older adults, those with diabetes or CKD, and those receiving potassium-raising medications; a 1 mg starting dose is recommended for patients considered at increased risk of hyperkalaemia or hyponatraemia.

Medication non-adherence is an important cause of pseudo-resistant HTN, as is under treatment, white coat effect and poor measurement technique [128]. These factors must be ameliorated before diagnosing resistant hypertension. Secondary hypertension is also an important contributor to uncontrolled/resistant HTN. Because these drugs (ASI) suppress aldosterone and increase renin, they are bound to significantly alter the Aldosterone renin ratio (ARR) in patients with hypertension. This has practical implications in a hypertension clinic where ARR may be utilised for evaluation of secondary hypertension. Hence, ARR must be estimated before initiating ASI therapy.

Finally, the role of ASIs in primary aldosteronism, particularly as a medical alternative to adrenalectomy in unilateral disease, represents a potentially transformative application that could fundamentally change the management paradigm for this condition [93]. If Phase III data confirm that ASIs can effectively suppress aldosterone secretion in patients with unilateral aldosterone-producing adenomas, reducing blood pressure and reversing hypokalaemia to a degree comparable to surgical cure, this would represent a major advance in the management of the most common form of secondary hypertension worldwide. The imminent Phase III development of baxdrostat in primary aldosteronism, and the Phase II data already generated for dexfadrostat in this indication, provide the evidentiary foundation for this investigational direction. The answers to these questions, anticipated over the next five to seven years, will determine whether ASIs fulfil their considerable mechanistic promise and establish themselves as an important agent in cardiorenal pharmacotherapy in the twenty-first century.

## 6. Conclusions

Aldosterone excess plays a central role in resistant hypertension and cardiorenal disease progression. While current RAAS-targeted therapies improve outcomes, their effectiveness is limited by aldosterone breakthrough, persistent non-genomic aldosterone effects, hyperkalaemia, and off-target adverse effects. Second-generation aldosterone synthase inhibitors have overcome many limitations of earlier compounds through improved CYP11B2 selectivity, enabling effective aldosterone suppression without clinically significant cortisol inhibition. Recent clinical trials have demonstrated meaningful reductions in blood pressure and albuminuria with generally acceptable safety profiles, although hyperkalaemia remains the principal class-related adverse effect. Ongoing cardiovascular and renal outcome trials will determine the long-term clinical role of ASIs in resistant hypertension, chronic kidney disease, heart failure, and primary aldosteronism.

## Figures and Tables

**Table 1:** Overview of aldosterone synthase inhibitors highlighting mechanisms of action, clinical development, safety findings, and major therapeutic outcomes in hypertension and cardiorenal disease.

Drug Name (Generic/Code)	Mechanism of Action	Phase Completed	Phase Ongoing	Adverse Effects Observed	Primary Endpoints	Key Results
First-Generation Aldosterone Synthase Inhibitors						
**FAD286** (R-fadrozole)	CYP11B2 inhibitor (dextro-enantiomer of fadrozole/aromatase inhibitor CGS16949). Preclinical only; tested in animal models of hypertension, HF, and CKD. Minimal aromatase activity	Preclinical only (No human Phase completed)	None (discontinued)	Severe dehydration & hyperkalaemia when combined with spironolactone; intestinal inflammation aggravation in DSS rat model (colonic aldosterone paradoxically increased)	Aldosterone reduction, BP lowering, organ fibrosis prevention in animal models	Dose-dependent aldosterone reduction (~50% adrenal); prevented cardiac hypertrophy & fibrosis. Not as effective as ARB for BP. Discordant enantioselective anti-fibrotic effect. Not advanced to human trials due to safety concerns in combination therapy.
**LCI699 ** **(Osilodrostat) ** **(Isturisa^®^)**	First human-tested oral ASI; derived structurally from FAD286. Inhibits CYP11B2 (aldosterone synthase) potently; also inhibits CYP11B1 (11β-hydroxylase) at higher doses → cortisol reduction FDA/EMA approved for Cushing’s disease (2020)	Phase II (hypertension, PA); Phase III (Cushing’s disease—LINC 3 & LINC 4, completed)	None (repurposed to Cushing’s disease; no ongoing HTN trials)	Adrenal insufficiency (51% in LINC 3, partly due to aggressive titration); fatigue; nausea/vomiting; headache; oedema; hypokalaemia; accumulation of 11-deoxycorticosterone (DOC, +710–1427% at low doses); blunted cortisol response to ACTH; hypertension (from DOC mineralocorticoid activity); hirsutism/acne	Change in SBP from baseline; aldosterone reduction; UFC normalisation (Cushing’s)	Dose-dependent aldosterone reduction (up to 80%) and SBP lowering in HTN/PA studies; potassium normalised in PA. However, excessive DOC accumulation and 11β-hydroxylase off-target effects halted HTN development. Repurposed successfully for Cushing’s disease (FDA approved 2020). Real-world HTN data (*n* = 80, TriNetX): BP control rose from 16% at 4 wks to 60% at 52 wks; adverse events stable ~12.5%.
**LY3045697**	More selective CYP11B2 inhibitor than LCI699. Non-imidazole structure. Greater selectivity for aldosterone synthase vs. 11β-hydroxylase compared to LCI699, but limited potency at higher doses	Phase II (two RCTs completed, c. 2014–2016)	None (development discontinued)	Limited potency at higher doses required; dose-related electrolyte disturbances (hyperkalaemia); cortisol-axis effects at supratherapeutic doses	Aldosterone reduction; BP lowering in hypertension	Greater CYP11B2 selectivity vs. LCI699 confirmed. However, extended use revealed inadequate potency higher doses needed for equivalent aldosterone suppression, compromising tolerability. Development discontinued.
Second-Generation Aldosterone Synthase Inhibitors						
**Baxdrostat ** **(CIN-107/RO6836191) ** **(AstraZeneca )**	Highly selective, potent oral CYP11B2 inhibitor (tetrahydroisoquinoline structure). Selectivity ratio CYP11B1/CYP11B2 = 100:1. Once-daily dosing (t^1/2^ ~ 29–30 h). Does not inhibit cortisol synthesis at therapeutic doses	Phase I (PK/PD, renal function cohorts) Phase II: BrigHTN (rHTN, 2022), HALO (uHTN, 2022–23), SPARK (PA, 2024) Phase III: BaxHTN (completed 2025), Bax24 (completed 2025)	Phase III ongoing: BaxAsia (Asia-Pacific HTN) FigHTN-CKD (CKD + HTN) PA Phase III (planned Aug 2025) Heart failure prevention trial Dapagliflozin + baxdrostat combination (CKD)	Mild-moderate: Hyperkalaemia (dose-related, serum K^+^ > 5.0 mmol/L, no severe cases in Phase II/III); hyponatraemia (mild); transient eGFR decrease (reversible); hypotension requiring intervention (rare). No adrenocortical insufficiency reported. No cortisol effects. SAEs: only 2/248 in HALO; generally well tolerated. Favourable safety in renal impairment	Primary: Change in seated SBP from baseline to Week 12 vs. placebo Secondary: DBP change; SBP <130 mmHg at Wk 12; 24 h ambulatory BP (Bax24); SBP after randomised withdrawal (Wk 24–32)	BrigHTN (Ph2, *n* = 248, rHTN): Dose-dependent SBP reduction; 1 mg & 2 mg significantly reduced SBP vs. placebo (~11 mmHg placebo-adjusted at 2 mg); aldosterone suppressed; no cortisol effect; no adrenal insufficiency. HALO (Ph2, uHTN): No significant SBP reduction vs. placebo (possible improved adherence in placebo arm); aldosterone reduced across all doses. SPARK (Ph2a, *n* = 15, PA, 2024): Baxdrostat resolved or reduced hypertension, excess aldosterone, and hypokalaemia in PA. BaxHTN (Ph3, *n* = 796, 2025): MET PRIMARY + ALL SECONDARY ENDPOINTS. ~10 mmHg placebo-adjusted SBP reduction at Wk 12 (both 1 mg & 2 mg). Favourable safety. Published NEJM Aug 2025. Bax24 (Ph3, *n* = 218, rHTN, 2025): MET PRIMARY ENDPOINT—highly clinically meaningful 24 h ambulatory SBP reduction. Top-line Oct 2025.
**Lorundrostat ** **(MLS-101) ** **(Mineralys Therapeutics)**	Highly selective oral CYP11B2 inhibitor. Spares CYP11B1 (11β-hydroxylase)— no cortisol suppression. Once-daily oral administration. Reduces aldosterone via direct enzymatic block in zona glomerulosa	Phase I Phase II: TARGET-HTN (2023), Advance-HTN (2025) Phase II: Explore-CKD (2025) Phase III: Launch-HTN (completed 2025)	Phase III ongoing/planned: EXPLORE-OSA (obstructive sleep apnea + HTN; data anticipated in Q1 2026) NDA submission planned to FDA Explore-CKD long-term extension	Hyperkalaemia (pooled RR 7.93; principal dose-limiting AE; generally mild, manageable); hyponatraemia (RR 1.96); hypotension (RR 3.06); any AE (RR 1.47). No adrenal insufficiency (CYP11B1-sparing confirmed). No cortisol suppression. Low rates of serious AEs. Meta-analysis (1562–1568 pts, 3 RCTs): AEs low-level and tolerable in Launch-HTN Ph3	Primary: Change in automated office SBP at Week 6 (Launch-HTN); SBP change at Week 12 (TARGET-HTN); 24 h ambulatory SBP change Secondary: DBP; UACR reduction (CKD); proportion achieving SBP targets	TARGET-HTN [82]: Dose-related SBP reduction averaging >14 mmHg at clinic visits; primarily mild short-term hyperkalaemia. Advance-HTN [99]: Significant 24 h SBP reductions at 12 weeks in uncontrolled/resistant HTN populations. Launch-HTN (Ph3, JAMA Aug 2025): MET PRIMARY ENDPOINT—placebo-adjusted SBP reduction of ~9.1 mm Hg (*p* < 0.001) at Wk 6; consistent benefit in uncontrolled and resistant HTN (*n* = 1083, 13 countries). NDA planned. Explore-CKD (Ph2, ASN Nov 2025): MET PRIMARY ENDPOINT: 9.3 mmHg office SBP reduction; 25.6% placebo-adjusted UACR reduction (*p* = 0.0015) first ASI to demonstrate albuminuria reduction in CKD.
**Dexfadrostat Phosphate ** **(DP-13) ** **(Idorsia/Recordati)**	Highly selective CYP11B2 inhibitor derived via proprietary enantioselective crystallisation (99.9% enantiomeric excess) from fadrozole’s dextro-enantiomer. Minimal CYP11B1 inhibition. Oral, once daily. Suppresses aldosterone-to-renin ratio (ARR)	Phase I (healthy volunteers, dose-escalation) Phase II (PA, completed 2022; published 2024)	Further phase development status unclear post-Idorsia restructuring (2024); no registered Phase III as of March 2026	Generally well tolerated; dose-dependent mild increases in corticosterone; mild ARR suppression-related effects. No adrenal insufficiency reported in Phase II. No significant cortisol effects at doses 4–12 mg. Mild headache, dizziness reported at higher doses	Primary: Change in ARR (aldosterone-to-renin ratio); SBP change from baseline Secondary: Aldosterone suppression; potassium normalisation; safety & tolerability	Phase I (healthy volunteers, Mulatero 2023 [108]): Dose-dependent aldosterone/ARR suppression with minimal corticosterone impact; confirmed CYP11B2 selectivity. Phase II PA (Mulatero et al. [109] NCT04007406, *n* = ~90, 8 wks, doses 4/8/12 mg): Significant ARR reduction and SBP lowering. Favourable safety. No Phase III registered to date; development trajectory uncertain.
**Vicadrostat ** **(BI 690517) ** **(Boehringer Ingelheim/Lilly)**	Potent, selective oral CYP11B2 inhibitor. Does not inhibit CYP11B1; cortisol levels unaffected. Specifically targeted at CKD and heart failure indications. Being studied in combination with empagliflozin (SGLT2i) to mitigate hyperkalaemia risk and exploit synergistic organ-protective effects	Phase I (4 studies: European SRD, Chinese/Japanese SRD, European MRD, Japanese MRD—completed 2017–2020) Phase II CKD (NCT05182840, Lancet 2024—completed)	Phase III: EASi-KIDNEY (NCT06531824)—CKD outcome trial (ongoing, ~1070 events/stratum, median 3 yr follow-up) EASi-HF Preserved (NCT06424288)—HFpEF (~6000 pts) EASi-HF Reduced (NCT06935370)—HFrEF	Phase I: Generally well tolerated; drug-related AEs: European SRD 8.3%; Chinese/Japanese SRD 21.4%; European MRD 13.9%; Japanese MRD 2.8%. Phase II CKD: Hyperkalaemia (dose-related risk, mitigated by background empagliflozin); mild AEs; no adrenal insufficiency; no cortisol suppression confirmed by LC-MS/MS corticosteroid metabolite analysis	Phase II Primary: Change in UACR (albuminuria) from baseline Phase III EASi-KIDNEY Primary: Time to first composite cardiorenal event (kidney failure, ≥40% eGFR decline, CV death, or hospitalisation for HF)	Phase I (2025 publication, Naunyn-Schmied): Dose-proportional PK; well tolerated across European and Asian populations; rapid oral absorption. Phase II CKD (Tuttle et al. [105], *n* = 586, albuminuric CKD): Vicadrostat 10 mg reduced UACR by ~40% vs. placebo; benefit seen with and without empagliflozin. Selective aldosterone suppression confirmed (cortisol unaffected). CYP11B2-selectivity confirmed by ASN 2025 post hoc LC-MS/MS analysis. Phase III EASi-KIDNEY underway; first large outcome trial for ASI class in CKD.

ACTH: Adrenocorticotropic hormone; AE: Adverse event; ARR: Aldosterone-to-renin ratio; ASI: Aldosterone synthase inhibitor; ASN: American Society of Nephrology; BP: Blood pressure; CKD: Chronic kidney disease; CV: Cardiovascular; CYP11B1: Steroid 11β-hydroxylase enzyme; CYP11B2: Aldosterone synthase enzyme; DBP: Diastolic blood pressure; DOC: 11-Deoxycorticosterone; DSS: Dextran sulfate sodium; eGFR: Estimated glomerular filtration rate; EMA: European Medicines Agency; FDA: Food and Drug Administration; HF: Heart failure; HFpEF: Heart failure with preserved ejection fraction; HFrEF: Heart failure with reduced ejection fraction; HTN: Hypertension; LC-MS/MS: Liquid chromatography–tandem mass spectrometry; MRD: Multiple rising dose; NEJM: New England Journal of Medicine; PA: Primary aldosteronism; PD: Pharmacodynamics; Ph: Phase; PK: Pharmacokinetics; RCT: Randomised controlled trial; rHTN: Resistant hypertension; SAE: Serious adverse event; SBP: Systolic blood pressure; SGLT2i: Sodium-glucose cotransporter-2 inhibitor; SRD: Single rising dose; t½: Elimination half-life; uHTN: Uncontrolled hypertension; UACR: Urinary albumin-to-creatinine ratio; UFC: Urinary free cortisol; Wk: Week.

## Data Availability

No new data were created or analyzed in this study. Data sharing is not applicable to this article.

## Abbreviations

The following abbreviations are used in this manuscript:

ACE	Angiotensin-converting enzyme
ACTH	Adrenocorticotropic hormone
AE	Adverse event
ARB	Angiotensin receptor blocker
ARR	Aldosterone-to-renin ratio
ASI	Aldosterone synthase inhibitor
AT1R	Angiotensin II type 1 receptor
CKD	Chronic kidney disease
CYP11B1	11β-Hydroxylase
CYP11B2	Aldosterone synthase
DBP	Diastolic blood pressure
DOC	11-Deoxycorticosterone
eGFR	Estimated glomerular filtration rate
ENaC	Epithelial sodium channel
HF	Heart failure
HFpEF	Heart failure with preserved ejection fraction
HFrEF	Heart failure with reduced ejection fraction
MRA	Mineralocorticoid receptor antagonist
MR	Mineralocorticoid receptor
PA	Primary aldosteronism
RAAS	Renin–angiotensin–aldosterone system
RH	Resistant hypertension
SBP	Systolic blood pressure
SGLT2	Sodium–glucose cotransporter 2
StAR	Steroidogenic acute regulatory protein
TGF-β	Transforming growth factor-beta
UACR	Urinary albumin-to-creatinine ratio
UFC	Urinary free cortisol
AT1	Angiotensin II type 1
MR	Mineralocorticoid receptors
CTGF	Connective tissue growth factor
ARB	Angiotensin receptor blockers
MRA	Mineralocorticoid receptor antagonists
